# Supplementary material for: ATR/Chk1 signaling induces autophagy through sumoylated RhoB-mediated lysosomal translocation of TSC2 after DNA damage
Source: Nat Commun. 2018 Oct 8;9:4139. doi: 10.1038/s41467-018-06556-9 (PMC6175864; doi:10.1038/s41467-018-06556-9)

Supplementary Information

ATR/Chk1 signaling induces autophagy through sumoylated RhoB-mediated lysosomal translocation of TSC2 after DNA damage

Liu et al

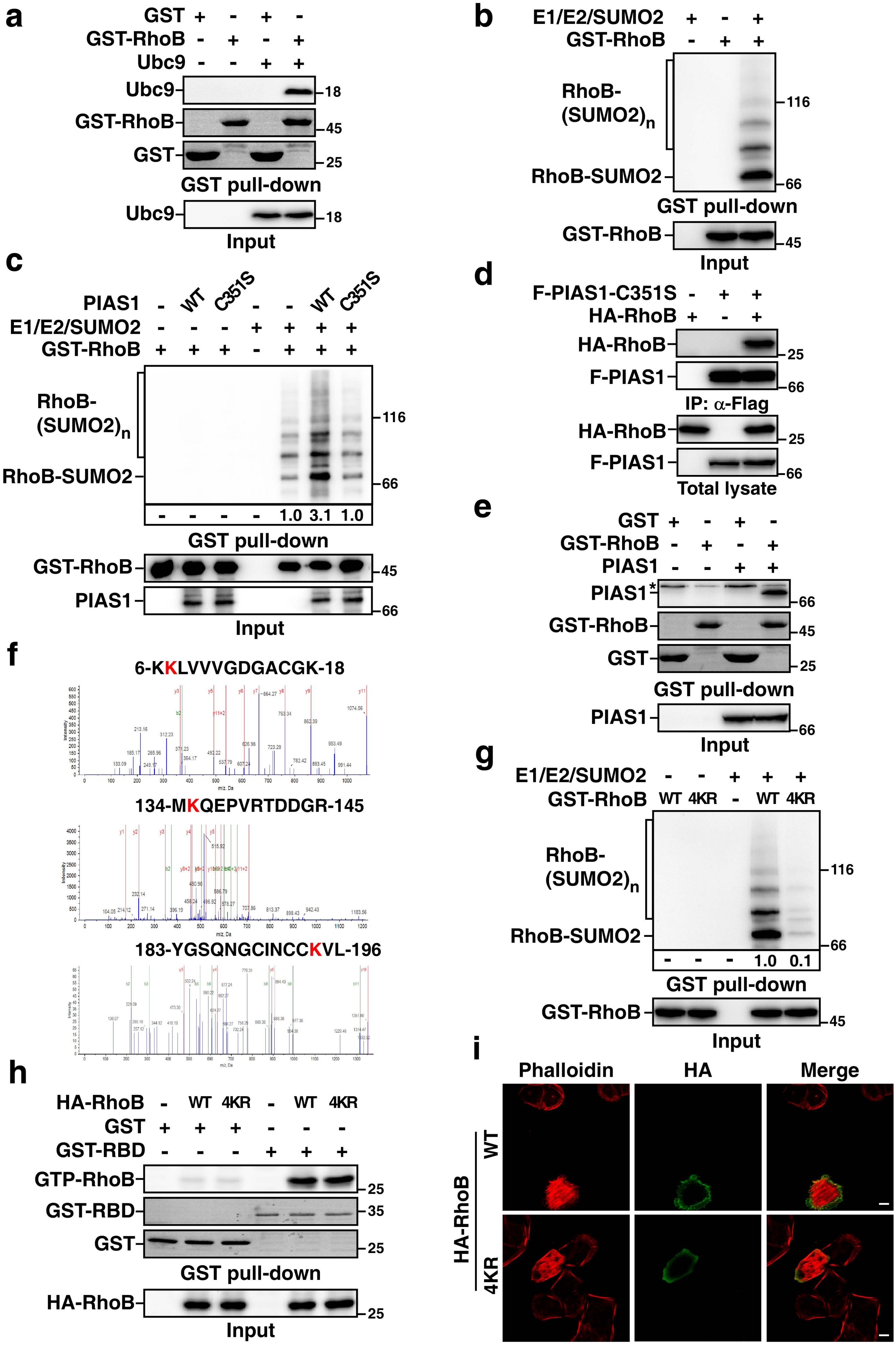

a
GST + - + -
GST-RhoB - + - +
Ubc9 - - + +
Ubc9
18
GST-RhoB
45
GST
25
GST pull-down
Ubc9
18
Input
b
E1/E2/SUMO2 + - +
GST-RhoB - + +
RhoB-(SUMO2)n
116
RhoB-SUMO2
66
GST pull-down
GST-RhoB
45
Input
c
PIAS1 - WT C351S - - WT C351S
E1/E2/SUMO2 - - - + + + +
GST-RhoB + + + - + + +
RhoB-(SUMO2)n
116
RhoB-SUMO2
66
- - - - 1.0 3.1 1.0
GST pull-down
GST-RhoB
45
PIAS1
66
Input
d
F-PIAS1-C351S - + +
HA-RhoB + - +
HA-RhoB
25
F-PIAS1
66
IP: α-Flag
HA-RhoB
25
F-PIAS1
66
Total lysate
e
GST + - + -
GST-RhoB - + - +
PIAS1 - - + +
PIAS1*
66
GST-RhoB
45
GST
25
GST pull-down
PIAS1
66
Input
f
6-KKLVVVGDGACGK-18
134-MKQEPVRTDDGR-145
183-YGSQNGCINCCKVL-196
g
E1/E2/SUMO2 - - + + +
GST-RhoB WT 4KR - WT 4KR
RhoB-(SUMO2)n
116
RhoB-SUMO2
66
- - - 1.0 0.1
GST pull-down
GST-RhoB
45
Input
h
HA-RhoB - WT 4KR - WT 4KR
GST + + + - - -
GST-RBD - - - + + +
GTP-RhoB
25
GST-RBD
35
GST
25
GST pull-down
HA-RhoB
25
Input
i
Phalloidin
HA
Merge
HA-RhoB
WT
4KR

Supplementary Figure 1. PIAS1 targets RhoB for sumoylation.

(a) *In vitro* interaction between Ubc9 and RhoB. Bacterially expressed and purified GST-tagged RhoB (GST-RhoB) and Ubc9 were subjected to GST pull-down assay. Associated Ubc9 was detected by immunoblotting assay.

(b) *In vitro* sumoylation of RhoB. Bacteria co-expressing with GST-RhoB, E1, Ubc9 (E2), and Myc-SUMO2 were lysed and subjected to GST pull-down assay followed by immunoblotting with anti-Myc to examine RhoB sumoylation.

(c) PIAS1 mediates sumoylation of RhoB *in vitro*. Bacteria co-expressing with GST-RhoB, E1, E2, Myc-SUMO2, and PIAS1 (WT or C351S) were subjected to *in vitro* sumoylation assay as described in (b).

(d) PIAS1 interacts with exogenous RhoB. HEK293T cells transfected with Flag-tagged PIAS1 catalytically inactive mutant (F-PIAS1 C351S) and/or HA-tagged RhoB (HA-RhoB) were subjected to anti-Flag immunoprecipitation (IP) followed by immunoblotting assay to detect associated RhoB.

(e) *In vitro* interaction between PIAS1 and RhoB. Bacterially expressed and purified GST-RhoB and PIAS1 were subjected to GST pull-down assay. Non-specific band is indicated with an asterisk.

(f) RhoB is sumoylated at the residues Lys7, Lys135, and Lys194 *in vitro*. Bacterially expressed GST-RhoB was applied to an *in vitro* sumoylation reaction and then subjected to mass spectrometry analysis to detect sumoylation sites of RhoB.

(g) RhoB-WT but not RhoB-4KR is sumoylated *in vitro*. Bacteria co-expressing E1, E2, Myc-SUMO2, and GST-RhoB (WT or 4KR) were subjected to *in vitro*

sumoylation assay as described in (b).

(h) The 4KR mutation does not affect GTP binding ability of RhoB. Bacterially expressed and purified GST-tagged RBD (GST-RBD) was incubated with cell lysate from HEK293T cells expressing HA-RhoB (WT or 4KR), and then subjected to GST pull-down assay to detect associated RhoB by Western blot analysis.

(i) RhoB-4KR promotes stress fiber formation. U2OS cells transfected with HA-RhoB (WT or 4KR) were subjected to immunofluorescence assay to visualize F-actin using Texas red conjugated Phalloidin. RhoB expression was determined with rat HA antibody. Scale bar, 10 μm.

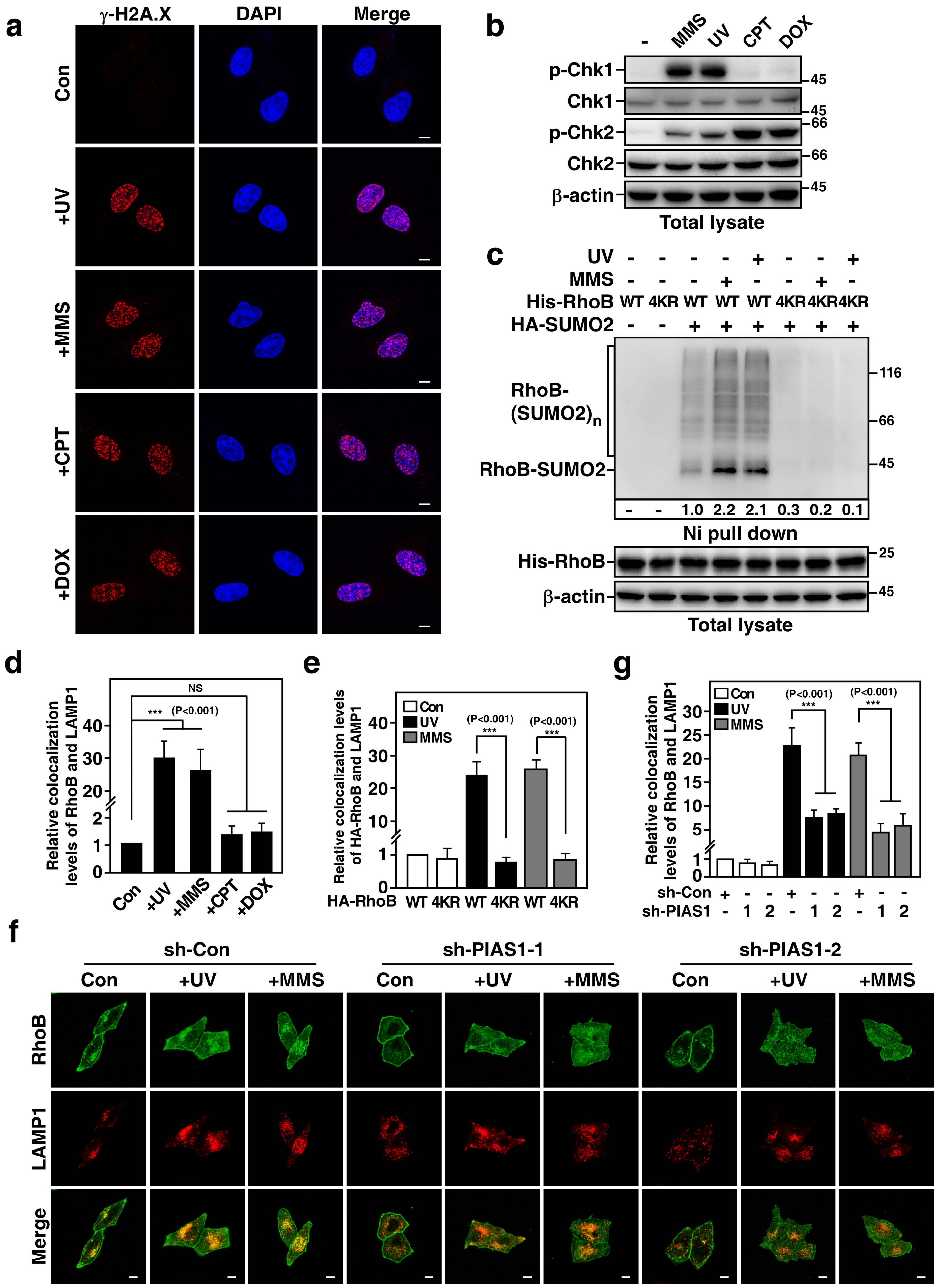
a
γ-H2A.X
DAPI
Merge
Con
+UV
+MMS
+CPT
+DOX
b
-
MMS
UV
CPT
DOX
p-Chk1
Chk1
p-Chk2
Chk2
β-actin
45
45
66
66
45
Total lysate
c
UV - - - - + - - +
MMS - - - + - - + -
His-RhoB WT 4KR WT WT WT 4KR 4KR 4KR
HA-SUMO2 - - + + + + + +
RhoB-(SUMO2)n
RhoB-SUMO2
116
66
45
- - 1.0 2.2 2.1 0.3 0.2 0.1
Ni pull down
His-RhoB
β-actin
25
45
Total lysate
d
Relative colocalization levels of RhoB and LAMP1
NS
*** (P<0.001)
Con
+UV
+MMS
+CPT
+DOX
e
Relative colocalization levels of HA-RhoB and LAMP1
Con
UV
MMS
(P<0.001)
***
HA-RhoB WT 4KR WT 4KR WT 4KR
g
Relative colocalization levels of RhoB and LAMP1
Con
UV
MMS
(P<0.001)
***
sh-Con + - - + - - + - -
sh-PIAS1 - 1 2 - 1 2 - 1 2
f
sh-Con
sh-PIAS1-1
sh-PIAS1-2
Con
+UV
+MMS
RhoB
LAMP1
Merge

Supplementary Figure 2. Sumoylation is required for translocation of RhoB to lysosomes.

(a) Treatment of UV, MMS, CPT, or DOX induces DNA damage. HeLa cells were subjected to immunofluorescence assay 1 hour after UV (80 Jm^{-2}) or 2 hours after MMS (0.5 mM), CPT (10 μM), or DOX (0.5 μM) treatment to examine levels of γ-H2A.X. Scale bar, 10 μm.

(b) UV or MMS treatment up-regulates phosphorylation levels of Chk1. HeLa cells treated as in (a) were subjected to immunoblotting assay to examine phosphorylation levels of Chk1 and Chk2.

(c) UV or MMS treatment enhances sumoylation of RhoB WT but not RhoB 4KR. Two hours after treated with MMS (0.5 mM) or 1 hour after treated with UV (80 Jm^{-2}), HEK293T cells expressing HA-SUMO2 and His-RhoB (WT or 4KR) were subjected to sumoylation assay followed by immunoblotting analysis to examine sumoylated RhoB.

(d) UV or MMS treatment enhances colocalization of RhoB and LAMP1. Colocalization levels of RhoB and LAMP1 was quantified using Imaris x64 image analysis software. Five random areas were counted for each experiment and data are presented as Mean ± SD of three individual experiments. One-way ANOVA ($F_{(4,70)}$=308.01) followed by LSD *post hoc* test for multiple comparisons.

(e) Wild-type RhoB but not RhoB-4KR co-localizes with LAMP1 in response to UV or MMS treatment. Colocalization of RhoB and LAMP1 was quantified as in (d). One-way ANOVA ($F_{(5,84)}$=896.1) followed by LSD *post hoc* test for multiple

comparisons.

(f, g) Knockdown of PIAS1 blocks lysosomal translocation of RhoB in response to UV or MMS treatment. HeLa cells transduced with expression of control shRNA (sh-Con) or shRNA against PIAS1 (sh-PIAS1-1 or sh-PIAS1-2) were subjected to immunofluorescence assay 2 hours after UV (80 Jm^{-2}) or 4 hours after MMS (0.5 mM) treatment to examine the localization of endogenous RhoB. Scale bar, 10 μm. Colocalization of RhoB and LAMP1 was quantified as in (d). One-way ANOVA ($F_{(8,126)}$=241.5) followed by LSD *post hoc* test for multiple comparisons.

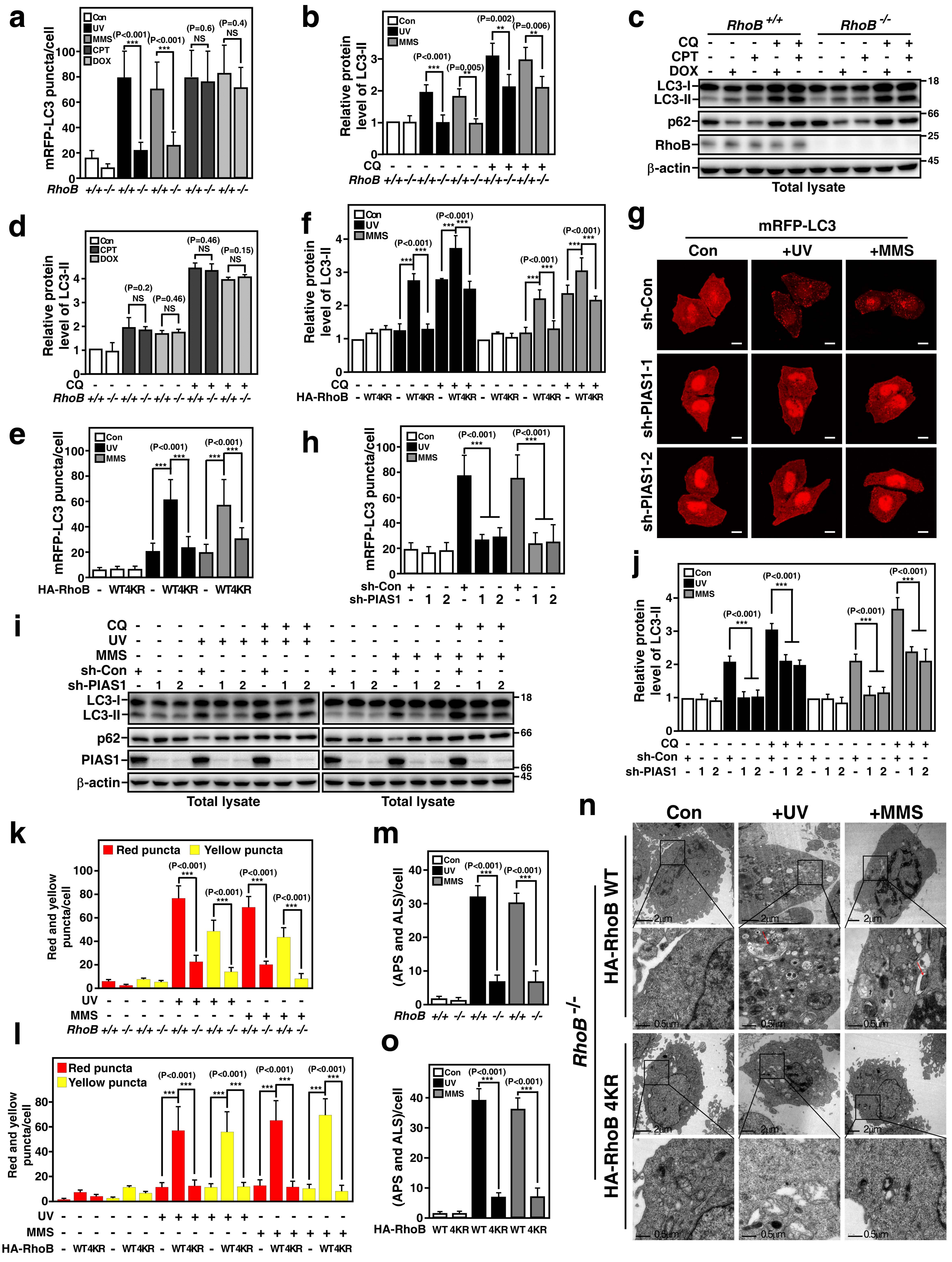
a
mRFP-LC3 puncta/cell
Con
UV
MMS
CPT
DOX
(P<0.001)
***
(P<0.001)
***
(P=0.6)
NS
(P=0.4)
NS
RhoB +/+ -/- +/+ -/- +/+ -/- +/+ -/- +/+ -/-
b
Relative protein level of LC3-II
Con
UV
MMS
(P<0.001)
***
(P=0.005)
**
(P=0.002)
**
(P=0.006)
**
CQ - - - - - - + + + +
RhoB +/+ -/- +/+ -/- +/+ -/- +/+ -/- +/+ -/-
c
RhoB +/+
RhoB -/-
CQ
CPT
DOX
LC3-I
LC3-II
p62
RhoB
β-actin
18
66
25
45
Total lysate
d
Relative protein level of LC3-II
Con
CPT
DOX
(P=0.2)
NS
(P=0.46)
NS
(P=0.46)
NS
(P=0.15)
NS
CQ - - - - - - + + + +
RhoB +/+ -/- +/+ -/- +/+ -/- +/+ -/- +/+ -/-
e
mRFP-LC3 puncta/cell
Con
UV
MMS
(P<0.001)
***
(P<0.001)
***
HA-RhoB - WT 4KR - WT 4KR - WT 4KR
f
Relative protein level of LC3-II
Con
UV
MMS
(P<0.001)
***
(P<0.001)
***
(P<0.001)
***
(P<0.001)
***
CQ
HA-RhoB - WT 4KR - WT 4KR - WT 4KR - WT 4KR - WT 4KR - WT 4KR
g
mRFP-LC3
Con
+UV
+MMS
sh-Con
sh-PIAS1-1
sh-PIAS1-2
h
mRFP-LC3 puncta/cell
Con
UV
MMS
(P<0.001)
***
(P<0.001)
***
sh-Con + - - + - - + - -
sh-PIAS1 - 1 2 - 1 2 - 1 2
i
CQ
UV
MMS
sh-Con
sh-PIAS1
LC3-I
LC3-II
p62
PIAS1
β-actin
18
66
66
45
Total lysate
Total lysate
j
Relative protein level of LC3-II
Con
UV
MMS
(P<0.001)
***
(P<0.001)
***
(P<0.001)
***
(P<0.001)
***
CQ
sh-Con
sh-PIAS1
k
Red and yellow puncta/cell
Red puncta
Yellow puncta
(P<0.001)
***
(P<0.001)
***
(P<0.001)
***
(P<0.001)
***
UV - - - - + + + + - - - -
MMS - - - - - - - - + + + +
RhoB +/+ -/- +/+ -/- +/+ -/- +/+ -/- +/+ -/- +/+ -/-
l
Red and yellow puncta/cell
Red puncta
Yellow puncta
(P<0.001)
***
(P<0.001)
***
(P<0.001)
***
(P<0.001)
***
UV
MMS
HA-RhoB - WT 4KR - WT 4KR - WT 4KR - WT 4KR - WT 4KR - WT 4KR
m
(APS and ALS)/cell
Con
UV
MMS
(P<0.001)
***
(P<0.001)
***
RhoB +/+ -/- +/+ -/- +/+ -/-
n
Con
+UV
+MMS
RhoB -/-
HA-RhoB WT
HA-RhoB 4KR
2μm
0.5μm
o
(APS and ALS)/cell
Con
UV
MMS
(P<0.001)
***
(P<0.001)
***
HA-RhoB WT 4KR WT 4KR WT 4KR

Supplementary Figure 3. Blockade of RhoB sumoylation impairs UV or MMS-induced autophagy

(a) Knockout of *RhoB* blocks UV or MMS-induced puncta formation of mRFP-LC3. The numbers of mRFP-LC3 puncta per cell were quantified using Imaris x64 image analysis software. Five random areas were counted for each experiment and data are presented as Mean ± SD of three individual experiments. One-way ANOVA ($F_{(9,140)}$=53.5) followed by LSD *post hoc* test for multiple comparisons.

(b) Knockout of *RhoB* diminishes augment of LC3-II induced by UV or MMS. Quantitative analysis of Western blot results is presented as Mean ± SD of three independent experiments. One-way ANOVA ($F_{(9,20)}$=19.653) followed by LSD *post hoc* test for multiple comparisons.

(c, d) Knockout of *RhoB* has no significant effect on CPT or DOX-induced augment of LC3-II. $RhoB^{+/+}$ or $RhoB^{-/-}$ cells pretreated 1 hour with or without 50 μM chloroquine (CQ) were subjected to immunoblotting assay 8 hours after treated with CPT (10 μM) or DOX (0.5 μM) (c). Quantification results are presented as Mean ± SD of three independent experiments. One-way ANOVA ($F_{(9,20)}$=257.1) followed by LSD *post hoc* test for multiple comparisons (d).

(e) Reintroduction of WT but not 4KR RhoB increases numbers of mRFP-LC3 puncta induced by UV or MMS treatment. The numbers of mRFP-LC3 puncta per cell were quantified as in (a). One-way ANOVA ($F_{(8,126)}$=60.28) followed by LSD *post hoc* test for multiple comparisons.

(f) WT but not 4KR RhoB induces augment of LC3-II after UV or MMS treatment. Quantitative analysis of immunoblotting assay results is presented as Mean ± SD of

three independent experiments. One-way ANOVA (UV: $F_{(8,18)}$=69.364; MMS: $F_{(8,18)}$=36.355) followed by LSD *post hoc* test for multiple comparisons.

(g, h) Knockdown of PIAS1 inhibits UV or MMS-induced puncta formation of mRFP-LC3. HeLa cells with stable expression of mRFP-LC3 and control shRNA (sh-Con) or shRNA against PIAS1 (sh-PIAS1-1 or sh-PIAS1-2) were subjected to immunofluorescence assay 4 hours after UV (80 Jm^{-2}) or 6 hours after MMS (0.5 mM) treatment. Scale bar, 10 μm (g). The numbers of mRFP-LC3 puncta per cell were quantified as in (a). One-way ANOVA ($F_{(8,126)}$=87.77) followed by LSD *post hoc* test for multiple comparisons (h).

(i, j) Knockdown of PIAS1 inhibits UV or MMS-induced augment of LC3-II. HeLa cells with stable expression of sh-Con, sh-PIAS1-1, or sh-PIAS1-2 were pretreated 1 hour with or without 50 μM chloroquine (CQ) and then subjected to immunoblotting assay 4 hours after treated with UV (80 Jm^{-2}) or 6 hours with MMS (0.5 mM) (i). Quantification of the immunoblotting assay is presented as Mean ± SD of three independent experiments. One-way ANOVA (UV: $F_{(8,18)}$=88.56; MMS: $F_{(8,18)}$=158.9) followed by LSD *post hoc* test for multiple comparisons (j).

(k) Knockout of *RhoB* decreases puncta formation of mRFP-GFP-LC3 induced by UV or MMS. The numbers of yellow or red puncta per cell were quantified using Imaris x64 image analysis software. Five random areas were counted for each experiment and data are presented as Mean ± SD of three individual experiments. One-way ANOVA (red puncta: $F_{(5,84)}$=332.89; yellow puncta: $F_{(5,84)}$=165.32) followed by LSD *post hoc* test for multiple comparisons.

(l) Reintroduction of WT but not 4KR RhoB restores puncta formation of

mRFP-GFP-LC3 induced by UV or MMS. The numbers of yellow or red puncta per cell were quantified as in (k). One-way ANOVA (red puncta: $F_{(8,126)}$=103.371; yellow puncta: $F_{(8,126)}$=161.4) followed by LSD *post hoc* test for multiple comparisons.

(m) Knockout of *RhoB* decreases the number of autophagosomes and autolysosomes. The numbers of autophagosomes (APS) and autolysosomes (ALS) per cell were quantified and plotted. Five random areas were counted for each experiment and data are presented as Mean ± SD of three individual experiments. One-way ANOVA ($F_{(5,84)}$=625.773) followed by LSD *post hoc* test for multiple comparisons.

(n, o) Reintroduction of WT but not 4KR RhoB increases UV or MMS-induced formation of autophagosomes and autolysosomes. Six hours after treated with UV (80 Jm^{-2}) or 8 hours after treated with MMS (0.5 mM) treatment, *RhoB*$^{-/-}$ cells with expression of HA-tagged WT or 4KR RhoB were assessed by electron microscopy. The magnified images are the areas indicated by the squares (n). Red arrow indicates autophagosome/autolysosome with mitochondria. The numbers of autophagosomes and autolysosomes per cell were quantified and plotted as in (m). One-way ANOVA ($F_{(5,84)}$=510.397) followed by LSD *post hoc* test for multiple comparisons (o).

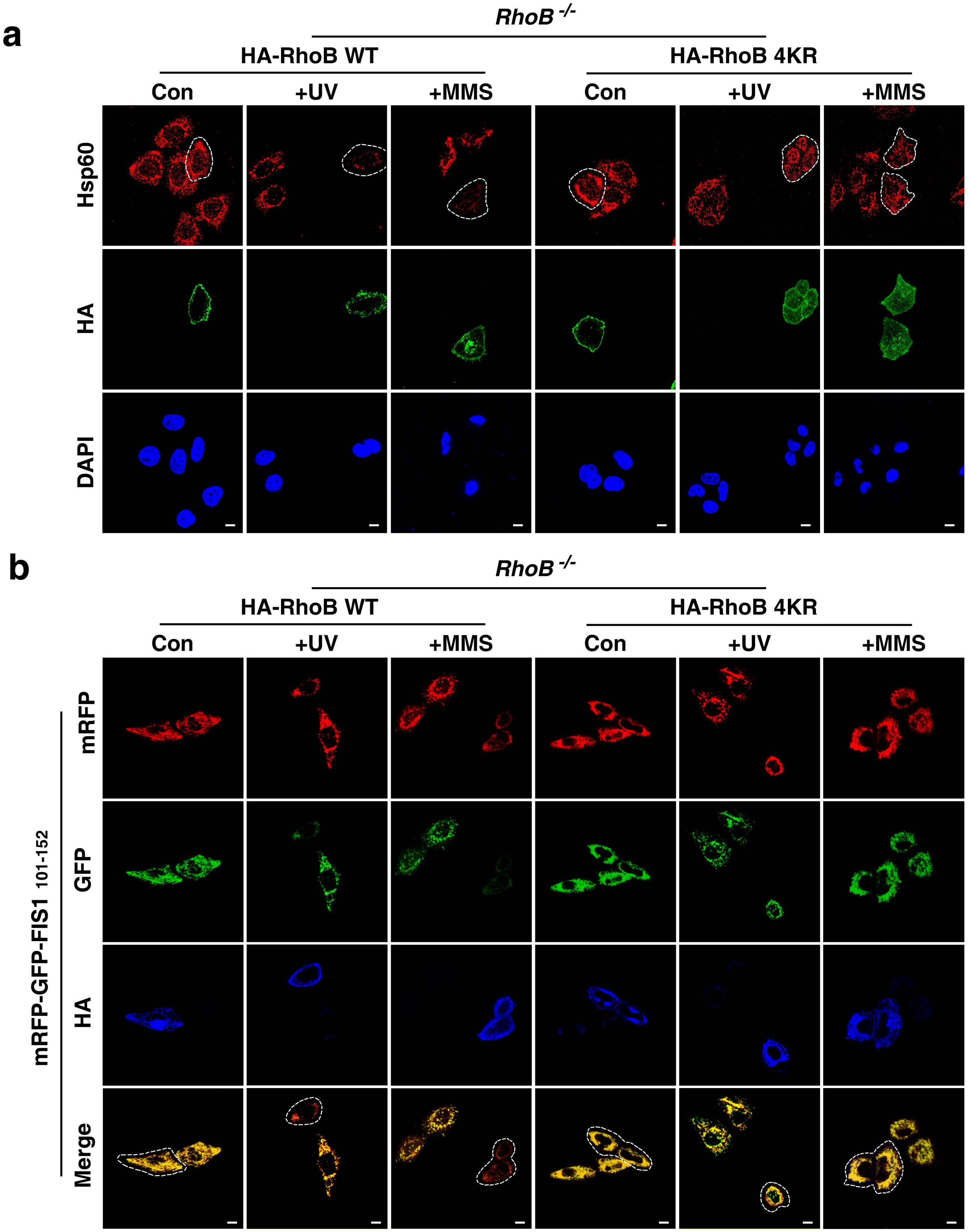

a
RhoB -/-
HA-RhoB WT
HA-RhoB 4KR
Con
+UV
+MMS
Con
+UV
+MMS
Hsp60
HA
DAPI
b
RhoB -/-
HA-RhoB WT
HA-RhoB 4KR
Con
+UV
+MMS
Con
+UV
+MMS
mRFP-GFP-FIS1 101-152
mRFP
GFP
HA
Merge

Supplementary Figure 4. Impeded RhoB sumoylation blocks UV or MMS-induced mitophagy

(a) Reintroduction of WT but not 4KR RhoB enhances the clearance of mitochondria induced by UV or MMS treatment. *RhoB*$^{-/-}$ cells transduced with lentivirus encoding HA-RhoB (WT or 4KR) were subjected to immunofluorescence assay 8 hours after treated with UV (80 Jm^{-2}) or 10 hours after treated with MMS (0.5 mM). The dot lines outline cells with expression of RhoB-WT or RhoB-4KR. Scale bar, 10 μm.

(b) Reintroduction of WT but not 4KR RhoB increases UV or MMS-promoted mitophagic flux. *RhoB*$^{-/-}$ cells with stable expression of mRFP-GFP-$FIS1_{101\text{-}152}$ were transduced with lentivirus encoding HA-RhoB (WT or 4KR) and subjected to fluorescence microscopy 8 hours after treated with UV (80 Jm^{-2}) or 10 hours after treated with MMS (0.5 mM). The dot lines outline cells with expression of WT or 4KR RhoB. Scale bar, 10 μm.

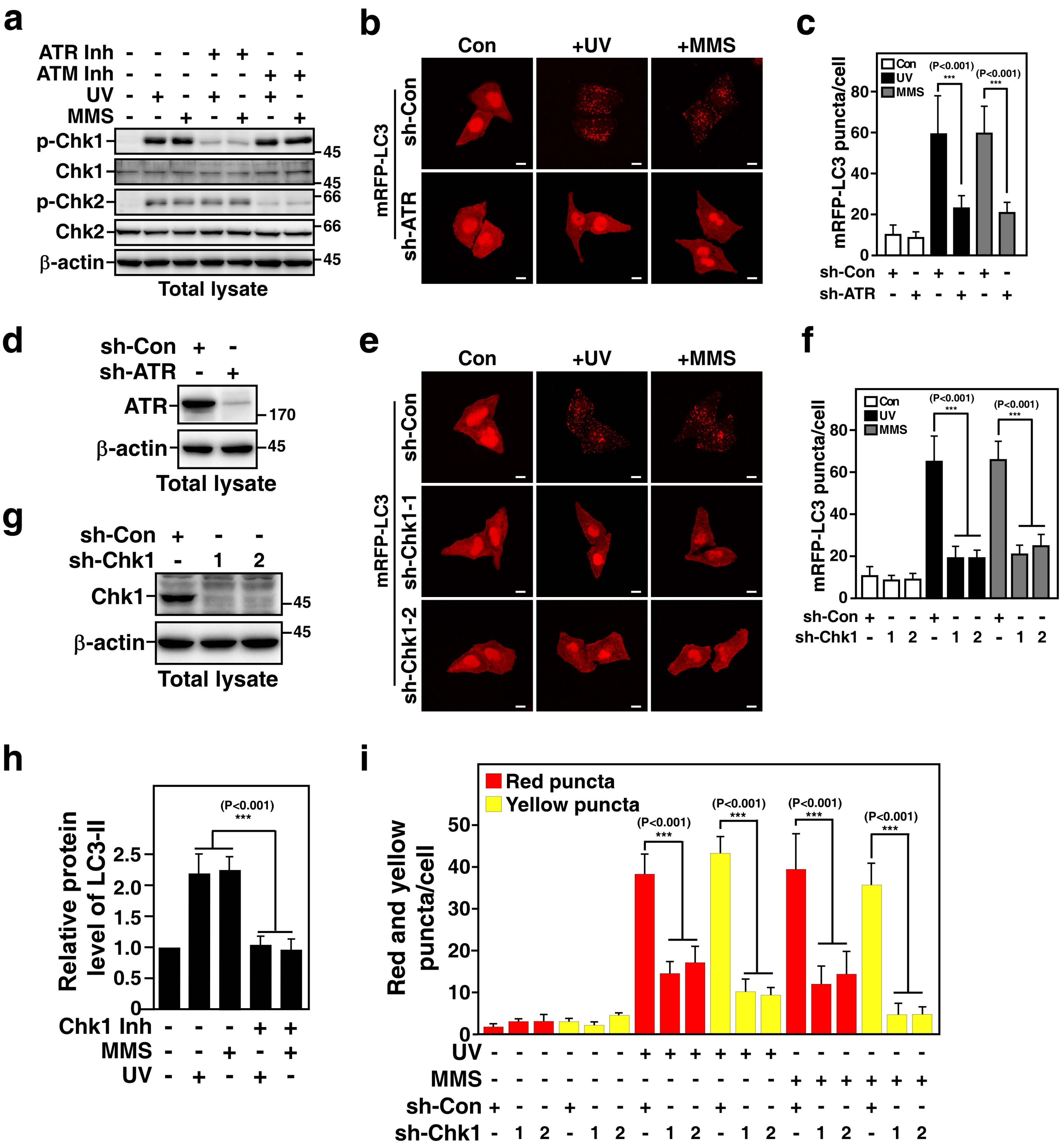

a
ATR Inh
ATM Inh
UV
MMS
p-Chk1
Chk1
p-Chk2
Chk2
β-actin
45
66
Total lysate
b
Con
+UV
+MMS
mRFP-LC3
sh-Con
sh-ATR
c
Con
UV
MMS
(P<0.001)
***
mRFP-LC3 puncta/cell
0
20
40
60
80
sh-Con
sh-ATR
d
sh-Con
sh-ATR
ATR
170
β-actin
45
Total lysate
e
Con
+UV
+MMS
mRFP-LC3
sh-Con
sh-Chk1-1
sh-Chk1-2
f
Con
UV
MMS
(P<0.001)
***
mRFP-LC3 puncta/cell
sh-Con
sh-Chk1
g
sh-Con
sh-Chk1
Chk1
β-actin
45
Total lysate
h
Relative protein level of LC3-II
0
0.5
1.0
1.5
2.0
2.5
(P<0.001)
***
Chk1 Inh
MMS
UV
i
Red puncta
Yellow puncta
Red and yellow puncta/cell
0
10
20
30
40
50
(P<0.001)
***
UV
MMS
sh-Con
sh-Chk1

Supplementary Figure 5. ATR/Chk1 signaling is critical for UV or MMS-induced autophagy

(a) ATR activity is required for UV or MMS-induced activation of Chk1. HeLa cells pretreated 0.5 hour with or without ATM inhibitor CP-466722 (10 μM) or ATR inhibitor VE-821 (1 μM) were subjected to immunoblotting assay 1 hour after UV (80 Jm^{-2}) or 2 hours after MMS (0.5 mM) treatment.

(b, c) Knockdown of ATR attenuates UV or MMS-induced puncta formation of mRFP-LC3. HeLa cells with stable expression of mRFP-LC3 and sh-Con or sh-ATR were subjected to immunofluorescence assay 4 hours after UV (80 Jm^{-2}) or 6 hours after MMS (0.5 mM) treatment. Scale bar, 10 μm (b). The numbers of red puncta per cell were determined using Imaris x64 image analysis software. Five random areas were counted for each experiment and data are presented as Mean ± SD of three individual experiments. One-way ANOVA ($F_{(5,84)}$=75.84) followed by LSD *post hoc* test for multiple comparisons (c).

(d) Knockdown efficiency of ATR. HeLa cells transduced with lentivirus encoding control shRNA (sh-Con) or shRNA against ATR (sh-ATR) were subjected to immunoblotting assay to examine the knockdown efficiency of ATR.

(e, f) Knockdown of Chk1 prevents UV or MMS-induced LC3 aggregation. HeLa cells with stable expression of mRFP-LC3 and sh-Con, sh-Chk1-1, or sh-Chk1-2 were treated and subjected to fluorescence microscopy as in (b). Quantification of red puncta per cell was as in (c). One-way ANOVA ($F_{(8,126)}$=190.4) followed by LSD *post hoc* test for multiple comparisons.

(g) Knockdown efficiency of Chk1. HeLa cells transduced with lentivirus encoding sh-Con or shRNA against Chk1 (sh-Chk1-1or sh-Chk1-2) were subjected to immunoblotting assay to examine the knockdown efficiency of Chk1.

(h) Chk1 activity is required for UV or MMS-induced accumulation of LC3-II. Quantitative analysis of Western blot results is presented as Mean ± SD of 3 independent experiments. One-way ANOVA ($F_{(4,10)}$=32.37) followed by LSD *post hoc* test for multiple comparisons.

(i) Knockdown of Chk1 inhibits UV or MMS-promoted autophagic flux. The numbers of yellow or red puncta per cell were quantified using Imaris x64 image analysis software. Five random areas were counted for each experiment and data are presented as Mean ± SD of three individual experiments. One-way ANOVA (red puncta: $F_{(8,126)}$=151.39; yellow puncta: $F_{(8,126)}$=397.03) followed by LSD *post hoc* test for multiple comparisons.

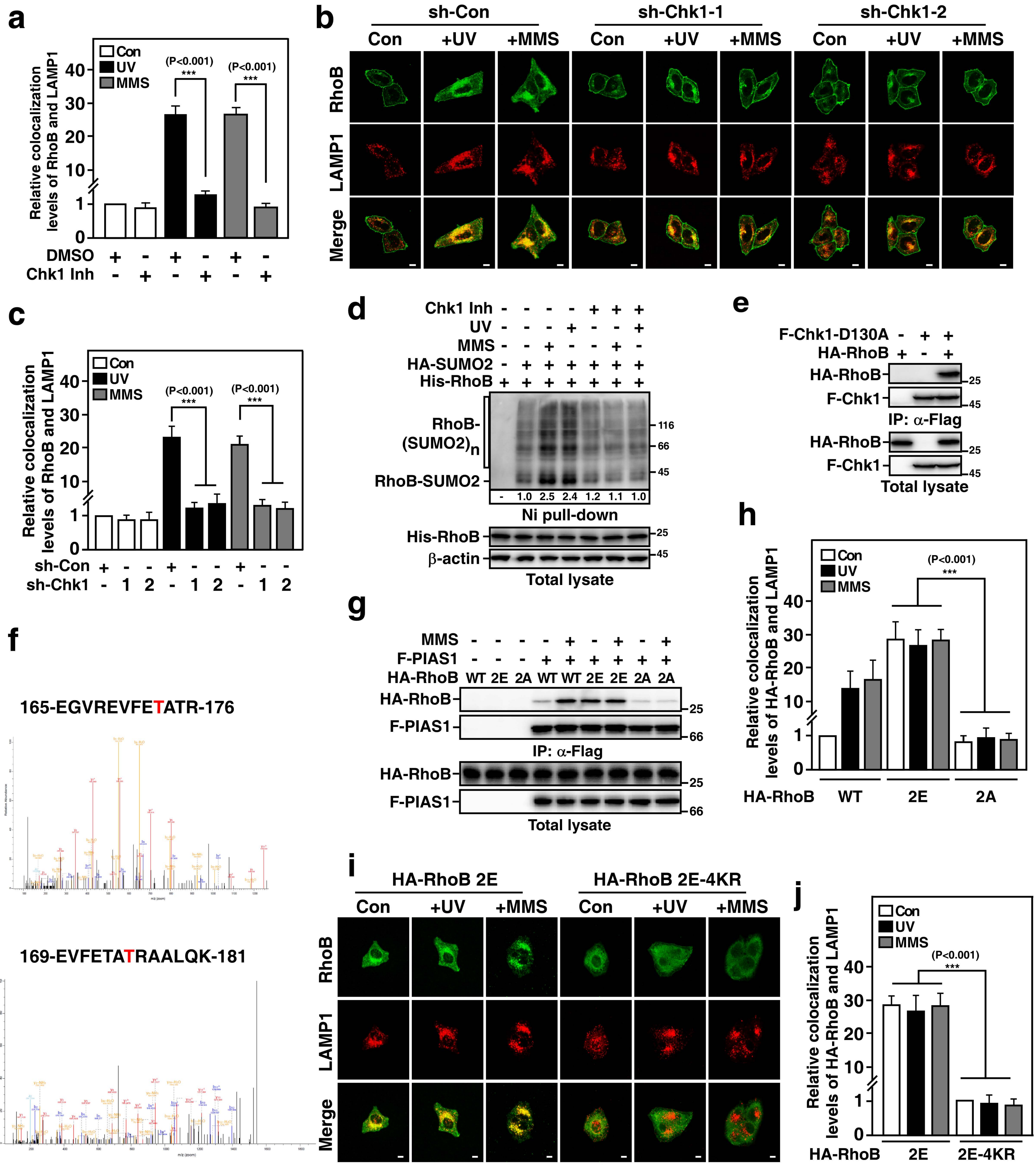

a
Con
UV
MMS
(P<0.001)
***
Relative colocalization levels of RhoB and LAMP1
DMSO
Chk1 Inh
b
sh-Con
sh-Chk1-1
sh-Chk1-2
Con
+UV
+MMS
RhoB
LAMP1
Merge
c
Relative colocalization levels of RhoB and LAMP1
sh-Con
sh-Chk1
d
Chk1 Inh
UV
MMS
HA-SUMO2
His-RhoB
RhoB-(SUMO2)n
RhoB-SUMO2
- 1.0 2.5 2.4 1.2 1.1 1.0
Ni pull-down
His-RhoB
β-actin
Total lysate
e
F-Chk1-D130A
HA-RhoB
F-Chk1
IP: α-Flag
Total lysate
f
165-EGVREVFETATR-176
169-EVFETATRAALQK-181
g
MMS
F-PIAS1
HA-RhoB WT 2E 2A WT WT 2E 2E 2A 2A
IP: α-Flag
Total lysate
h
Relative colocalization levels of HA-RhoB and LAMP1
HA-RhoB WT 2E 2A
i
HA-RhoB 2E
HA-RhoB 2E-4KR
j
HA-RhoB 2E 2E-4KR

Supplementary Figure 6. Chk1-mediated phosphorylation of RhoB is required for RhoB sumoylation and lysosomal translocation

(a) Treatment of Chk1 inhibitor blocks translocation of RhoB to lysosomes. Colocalization levels of RhoB and LAMP1 was quantified using Imaris x64 image analysis software. Five random areas were counted for each experiment and data are presented as Mean ± SD of three individual experiments. One-way ANOVA ($F_{(5,84)}$=1329.2) followed by LSD *post hoc* test for multiple comparisons.

(b, c) Knockdown of Chk1 prevents lysosomal translocation of RhoB. HeLa cells with stable expression of control shRNA (sh-Con) or shRNA against Chk1 (sh-Chk1-1 or sh-Chk1-2) were subjected to immunofluorescence assay 2 hours after UV (80 Jm^{-2}) or 4 hours after MMS (0.5 mM) treatment to examine the colocalization of endogenous RhoB and LAMP1. Scale bar, 10 μm (b). Colocalization of RhoB and LAMP1 was quantified as in (a). One-way ANOVA ($F_{(5,126)}$=721.1) followed by LSD *post hoc* test for multiple comparisons (c).

(d) Treatment with Chk1 inhibitor abolishes UV or MMS-promoted sumoylation of exogenous RhoB. HEK293T cells with expression of indicated combination of His-RhoB and HA-SUMO2 were pretreated with or without Chk1 inhibitor (0.2 μM), and then subjected to sumoylation assay 1 hour after UV (80 Jm^{-2}) or 2 hours after MMS (0.5 mM) treatment. SUMO2 conjugation of RhoB was detected by immunoblotting with HA antibody.

(e) Chk1 interacts with exogenous RhoB. HEK293T cells transfected with indicated combination of Flag-tagged Chk1 D130A mutant (F-Chk1 D130A) and HA-tagged

RhoB (HA-RhoB) were subjected to anti-Flag IP followed by immunoblotting with anti-HA to detect associated RhoB.

(f) Chk1 phosphorylates RhoB at the residues Thr173 and Thr175 *in vitro*. Phosphorylation sites of RhoB were detected by mass spectrometry analysis of bacterially expressed RhoB from an *in vitro* kinase reaction with Flag-tagged Chk1 immunoprecipitated from UV-treated HEK293T cells.

(g) Phosphorylation of RhoB by Chk1 promotes its binding to PIAS1. HEK293T cells with expression of indicated combination of Flag-tagged PIAS1 (F-PIAS1) and HA-tagged RhoB (WT, 2E, or 2A) were treated 2 hours with or without MMS (0.5 mM) before subjected to co-immunoprecipitation assay to detect associated RhoB.

(h) Phosphorylation of RhoB is essential for its lysosomal translocation. Colocalization of RhoB and LAMP1 was quantified as in (a). One-way ANOVA ($F_{(8,126)}$=189.9) followed by LSD *post hoc* test for multiple comparisons.

(i, j) Sumoylation is required for lysosomal translocation of RhoB-2E mutant. U2OS cells expressing HA-RhoB (2E or 2E-4KR) were subjected to immunofluorescence assay 4 hours after UV (80 Jm^{-2}) or MMS (0.5 mM) treatment to examine the localization of HA-RhoB and endogenous LAMP1. Scale bar, 10 μm (i). Colocalization of RhoB and LAMP1 was quantified as in (a). One-way ANOVA ($F_{(5,84)}$=425.4) followed by LSD *post hoc* test for multiple comparisons (j).

Figure S7

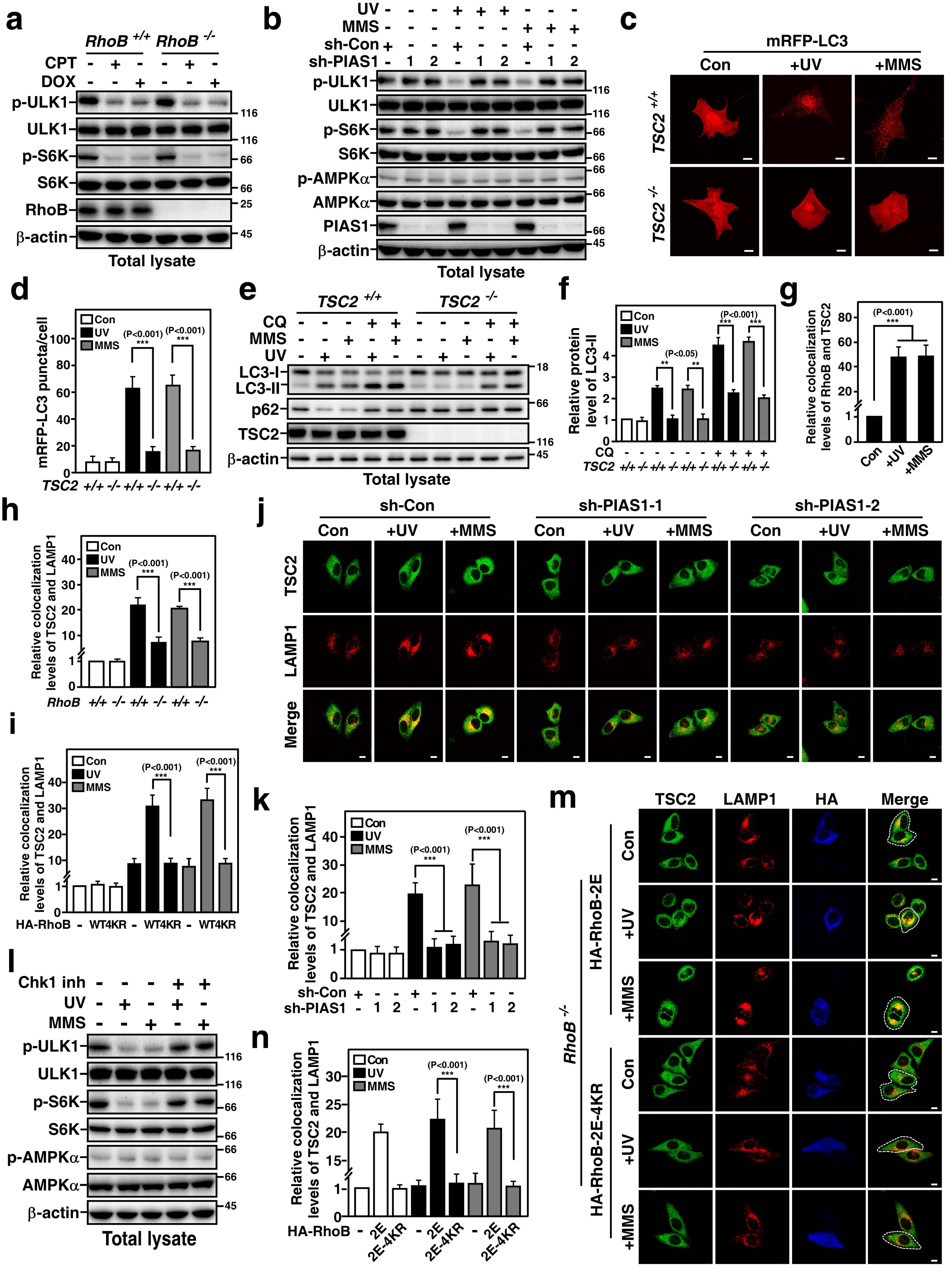
a
RhoB +/+ RhoB -/-
CPT
DOX
p-ULK1
ULK1
p-S6K
S6K
RhoB
β-actin
Total lysate
b
UV
MMS
sh-Con
sh-PIAS1
p-ULK1
ULK1
p-S6K
S6K
p-AMPKα
AMPKα
PIAS1
β-actin
Total lysate
c
mRFP-LC3
Con
+UV
+MMS
TSC2 +/+
TSC2 -/-
d
mRFP-LC3 puncta/cell
e
TSC2 +/+
TSC2 -/-
CQ
MMS
UV
LC3-I
LC3-II
p62
TSC2
β-actin
Total lysate
f
Relative protein level of LC3-II
g
Relative colocalization levels of RhoB and TSC2
h
Relative colocalization levels of TSC2 and LAMP1
j
sh-Con
sh-PIAS1-1
sh-PIAS1-2
TSC2
LAMP1
Merge
i
HA-RhoB
k
sh-Con
sh-PIAS1
l
Chk1 inh
UV
MMS
p-ULK1
ULK1
p-S6K
S6K
p-AMPKα
AMPKα
β-actin
Total lysate
m
TSC2
LAMP1
HA
Merge
RhoB -/-
HA-RhoB-2E
HA-RhoB-2E-4KR
n
HA-RhoB

Supplementary Figure 7. Both phosphorylation and sumoylation are required for RhoB-mediated recruitment of TSC2 to lysosomes

(a) Knockout of *RhoB* does not affect CPT or DOX-induced down-regulation of phosphorylated ULK1 and S6K. *RhoB*$^{+/+}$ or *RhoB*$^{-/-}$ cells were subjected to immunoblotting assay 6 hours after CPT (10 μM) or DOX (0.5 μM).

(b) Knockdown of PIAS1 inhibits down-regulation of phosphorylated ULK1 and S6K induced by UV or MMS. HeLa cells transduced with lentivirus encoding control shRNA (sh-Con) or shRNA against PIAS1 (sh-PIAS1-1 or sh-PIAS-2) were subjected to immunoblotting assay 2 hours after UV (80 Jm^{-2}) or 4 hours after MMS (0.5 mM).

(c, d) TSC2 is required for UV or MMS-induced mRFP-LC3 puncta formation. *TSC2*$^{+/+}$ or *TSC2*$^{-/-}$ cells with stable expression of mCherry red fluorescent protein fused LC3 (mRFP-LC3) were subjected to fluorescence microscopy to visualize mRFP-LC3 6 hours after UV (80 Jm^{-2}) or 5 hours after MMS (0.5 mM) treatment. Scale bar, 10 μm (c). The numbers of mRFP-LC3 puncta per cell were quantified using Imaris x64 image analysis software. Five random areas were counted for each experiment and data are presented as Mean ± SD of three individual experiments. One-way ANOVA ($F_{(5,84)}$=276) followed by LSD *post hoc* test for multiple comparisons (d).

(e, f) Knockout of *TSC2* blocks UV or MMS-induced augment of LC3-II. *TSC2*$^{+/+}$ or *TSC2*$^{-/-}$ cells pretreated 1 hour with or without 50 μM chloroquine (CQ) were subjected to immunoblotting assay 6 hours after treated with UV (80 Jm^{-2}) or 5 hours with MMS (0.5 mM) (e). Quantification results are presented as Mean ± SD of three independent experiments. One-way ANOVA (F(9,20)=1034.31) followed by LSD

post hoc test for multiple comparisons (f).

(g) Colocalization levels of RhoB and TSC2 are enhanced by UV or MMS treatment. Colocalization levels of TSC2 and RhoB was quantified using Imaris x64 image analysis software. Five random areas were counted for each experiment and data are presented as Mean ± SD of three individual experiments. One-way ANOVA ($F_{(2,42)}$=326.9) followed by LSD *post hoc* test for multiple comparisons.

(h) Knockout of *RhoB* inhibits UV or MMS-induced lysosomal translocation of TSC2. Colocalization levels of TSC2 and LAMP1 was quantified as in (g). One-way ANOVA ($F_{(5,84)}$=302.8) followed by LSD *post hoc* test for multiple comparisons.

(i) Wild-type RhoB but not RhoB-4KR promotes lysosomal translocation of TSC2. Colocalization levels of TSC2 and LAMP1 was quantified as in (g). One-way ANOVA ($F_{(8,126)}$=288.4) followed by LSD *post hoc* test for multiple comparisons.

(j, k) Knockdown of PIAS1 inhibits translocation of TSC2 to lysosomes in response to UV or MMS treatment. HeLa cells with stable expression of control shRNA (sh-Con) or shRNA against PIAS1 (sh-PIAS1-1 or sh-PIAS-2) were treated 2 hours with UV (80 Jm^{-2}) or 4 hours with MMS (0.5 mM) and then subjected to immunofluorescence assay to examine the localization of endogenous TSC2 and LAMP1. Scale bar, 10 μm (j). Colocalization levels of TSC2 and LAMP1 was quantified as in (g). One-way ANOVA ($F_{(8,126)}$=178.2) followed by LSD *post hoc* test for multiple comparisons (k).

(l) Chk1 activity is required for UV or MMS-induced down-regulation of phosphorylated ULK1 and S6K. HeLa cells pretreated pretreated 0.5 hour with or

without Chk1 inhibitor (0.2 μM) were treated another 2 hours with UV (80 Jm^{-2}) or 4 hours with MMS (0.5 mM) and then subjected to immunoblotting assay.

(m, n) Sumoylation is required for RhoB 2E mutant-mediated translocation of TSC2 to lysosomes. *RhoB*$^{-/-}$ cells transduced with lentivirus encoding HA-tagged RhoB (2E or 2E-4KR) and were treated and subjected to immunofluorescence assay as in (c). The dot lines outline cells with expression of HA-RhoB 2E or 2E-4KR. Scale bar, 10 μm (m). Colocalization levels of TSC2 and LAMP1 was quantified as in (g). One-way ANOVA ($F_{(8,126)}$=471.2) followed by LSD *post hoc* test for multiple comparisons (n).

Supplementary Figure 8 Uncropped blots with size marker indications.

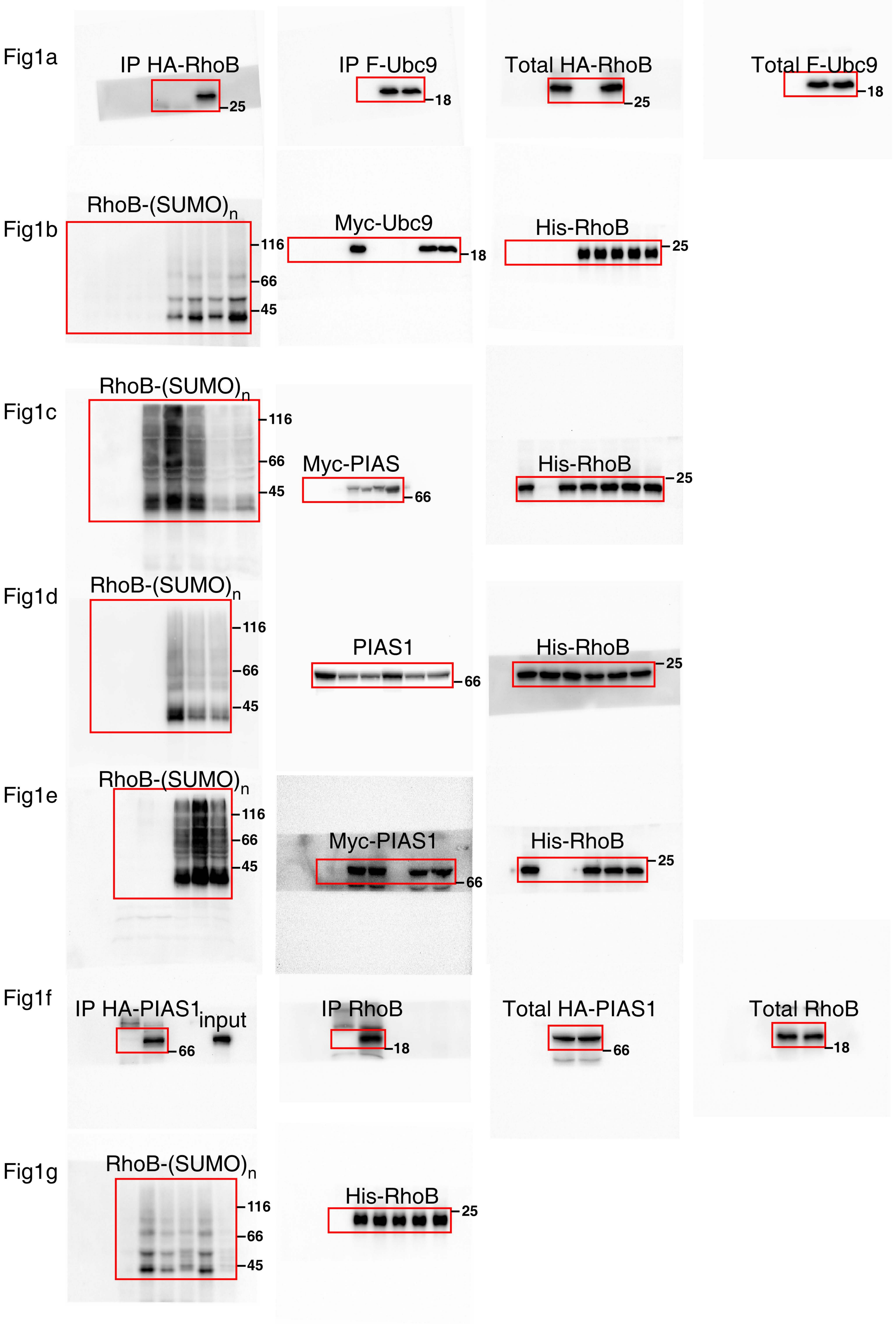

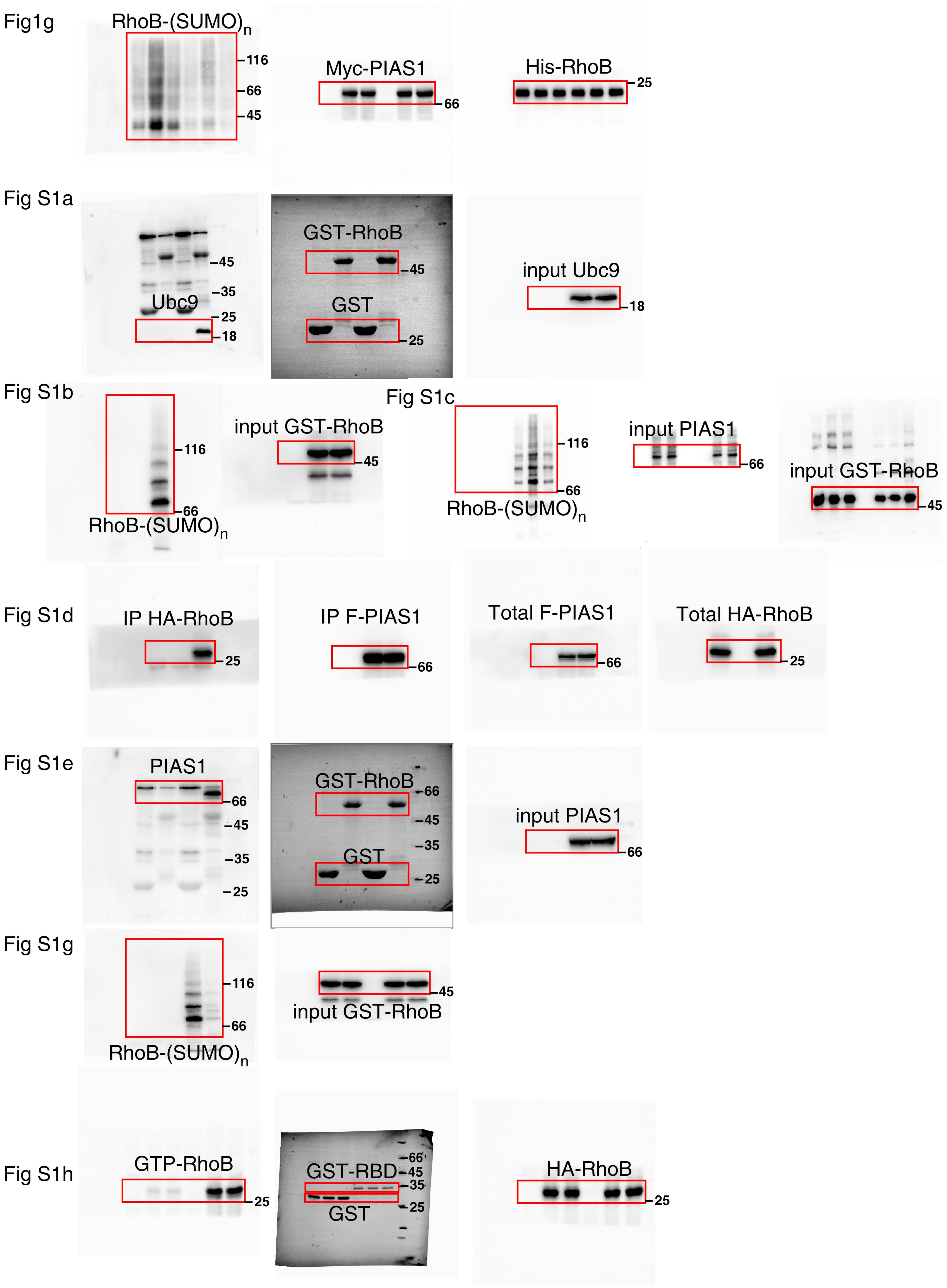

Fig1g
RhoB-(SUMO)n
116
66
45
Myc-PIAS1
66
His-RhoB
25
Fig S1a
45
35
Ubc9
25
18
GST-RhoB
45
GST
25
input Ubc9
18
Fig S1b
116
66
RhoB-(SUMO)n
input GST-RhoB
45
Fig S1c
116
66
RhoB-(SUMO)n
input PIAS1
66
input GST-RhoB
45
Fig S1d
IP HA-RhoB
25
IP F-PIAS1
66
Total F-PIAS1
66
Total HA-RhoB
25
Fig S1e
PIAS1
66
45
35
25
GST-RhoB
66
45
35
GST
25
input PIAS1
66
Fig S1g
116
66
RhoB-(SUMO)n
45
input GST-RhoB
Fig S1h
GTP-RhoB
25
GST-RBD
66
45
35
25
GST
HA-RhoB
25

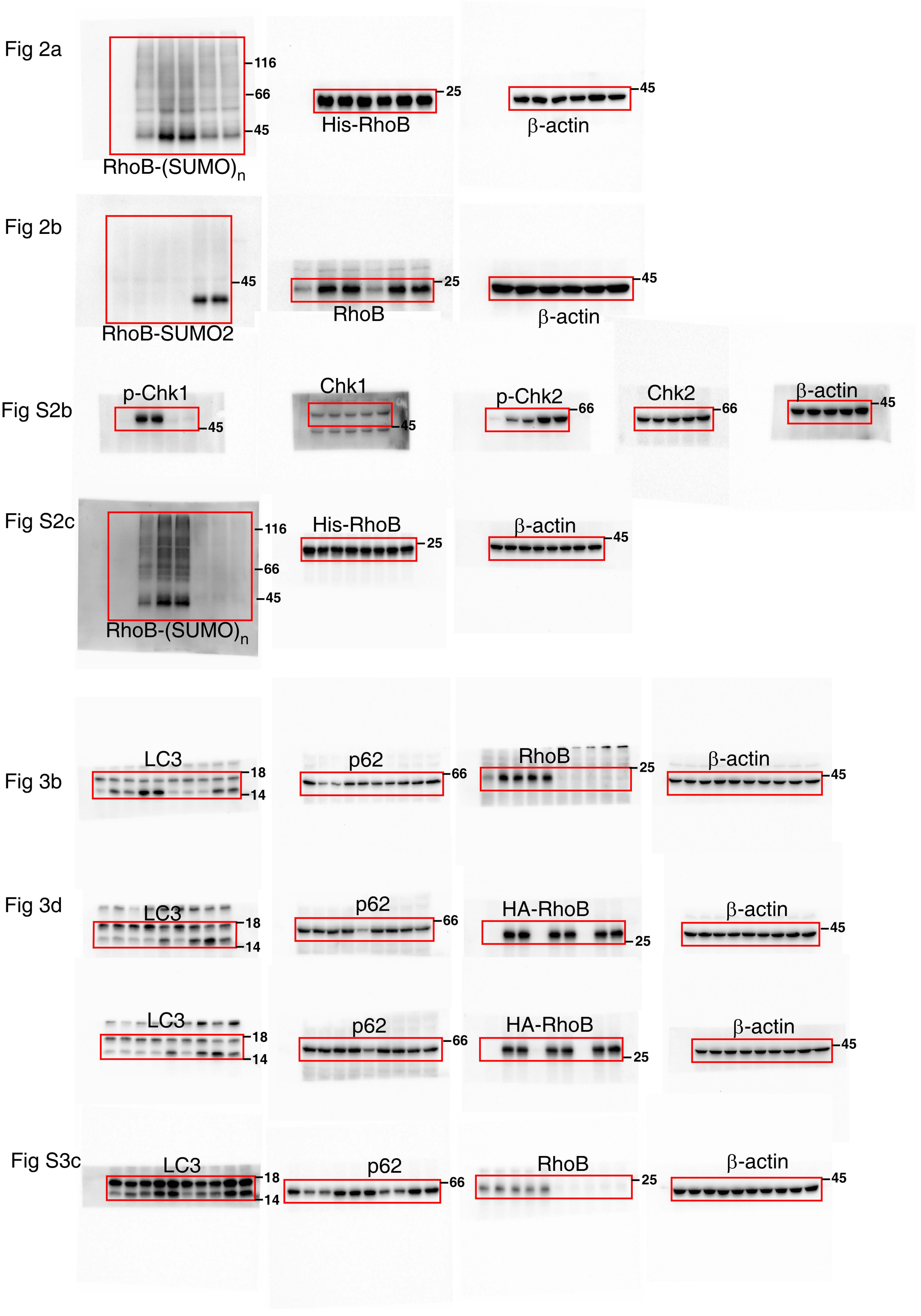

Fig 2a
116
66
45
RhoB-(SUMO)$_n$
25
His-RhoB
45
β-actin
Fig 2b
45
RhoB-SUMO2
25
RhoB
45
β-actin
Fig S2b
p-Chk1
45
Chk1
45
p-Chk2
66
Chk2
66
β-actin
45
Fig S2c
116
66
45
RhoB-(SUMO)$_n$
His-RhoB
25
β-actin
45
Fig 3b
LC3
18
14
p62
66
RhoB
25
β-actin
45
Fig 3d
LC3
18
14
p62
66
HA-RhoB
25
β-actin
45
LC3
18
14
p62
66
HA-RhoB
25
β-actin
45
Fig S3c
LC3
18
14
p62
66
RhoB
25
β-actin
45

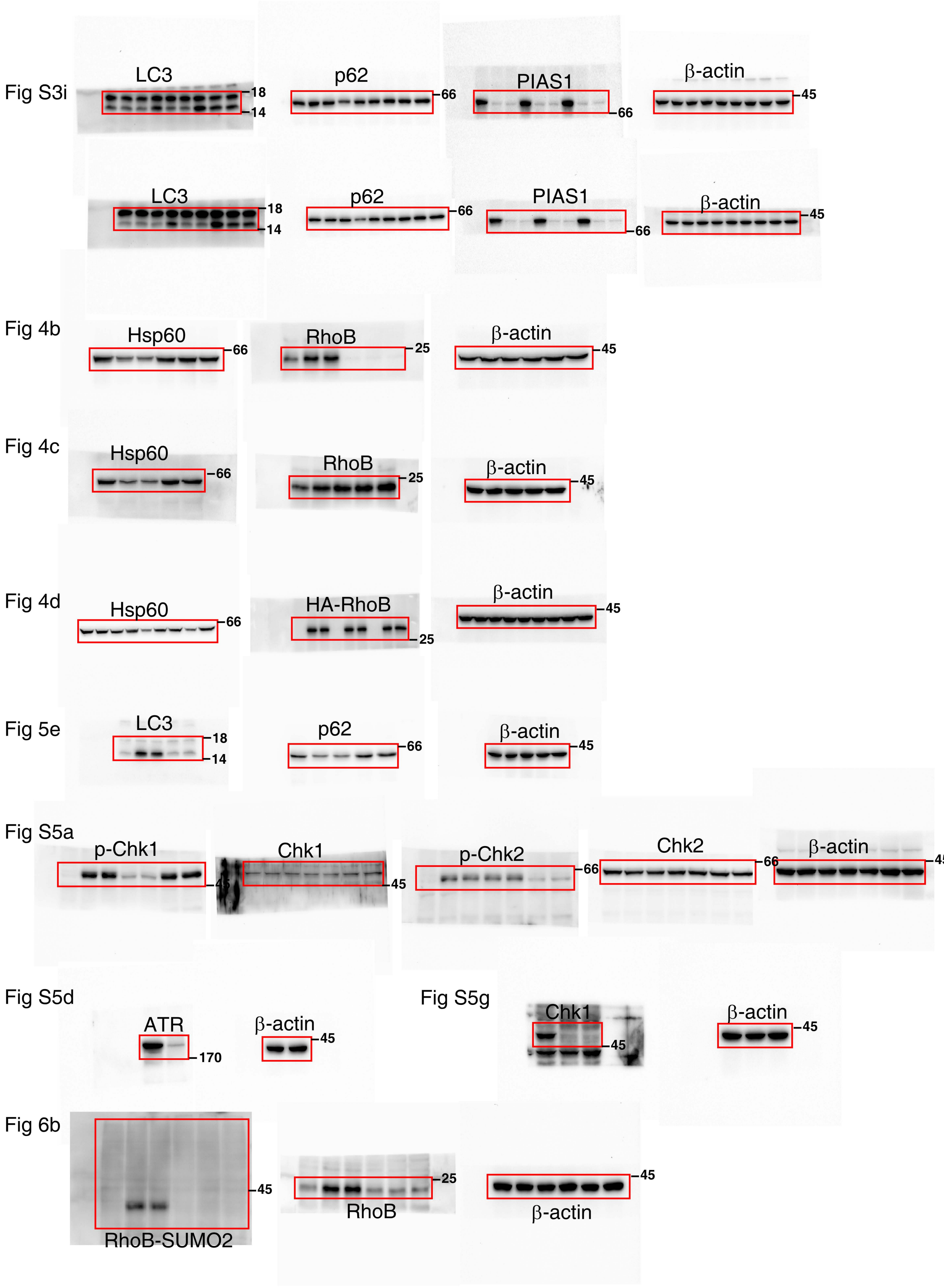
Fig S3i
LC3
18
14
p62
66
PIAS1
66
β-actin
45
LC3
18
14
p62
66
PIAS1
66
β-actin
45
Fig 4b
Hsp60
66
RhoB
25
β-actin
45
Fig 4c
Hsp60
66
RhoB
25
β-actin
45
Fig 4d
Hsp60
66
HA-RhoB
25
β-actin
45
Fig 5e
LC3
18
14
p62
66
β-actin
45
Fig S5a
p-Chk1
45
Chk1
45
p-Chk2
66
Chk2
66
β-actin
45
Fig S5d
ATR
170
β-actin
45
Fig S5g
Chk1
45
β-actin
45
Fig 6b
45
RhoB-SUMO2
25
RhoB
45
β-actin

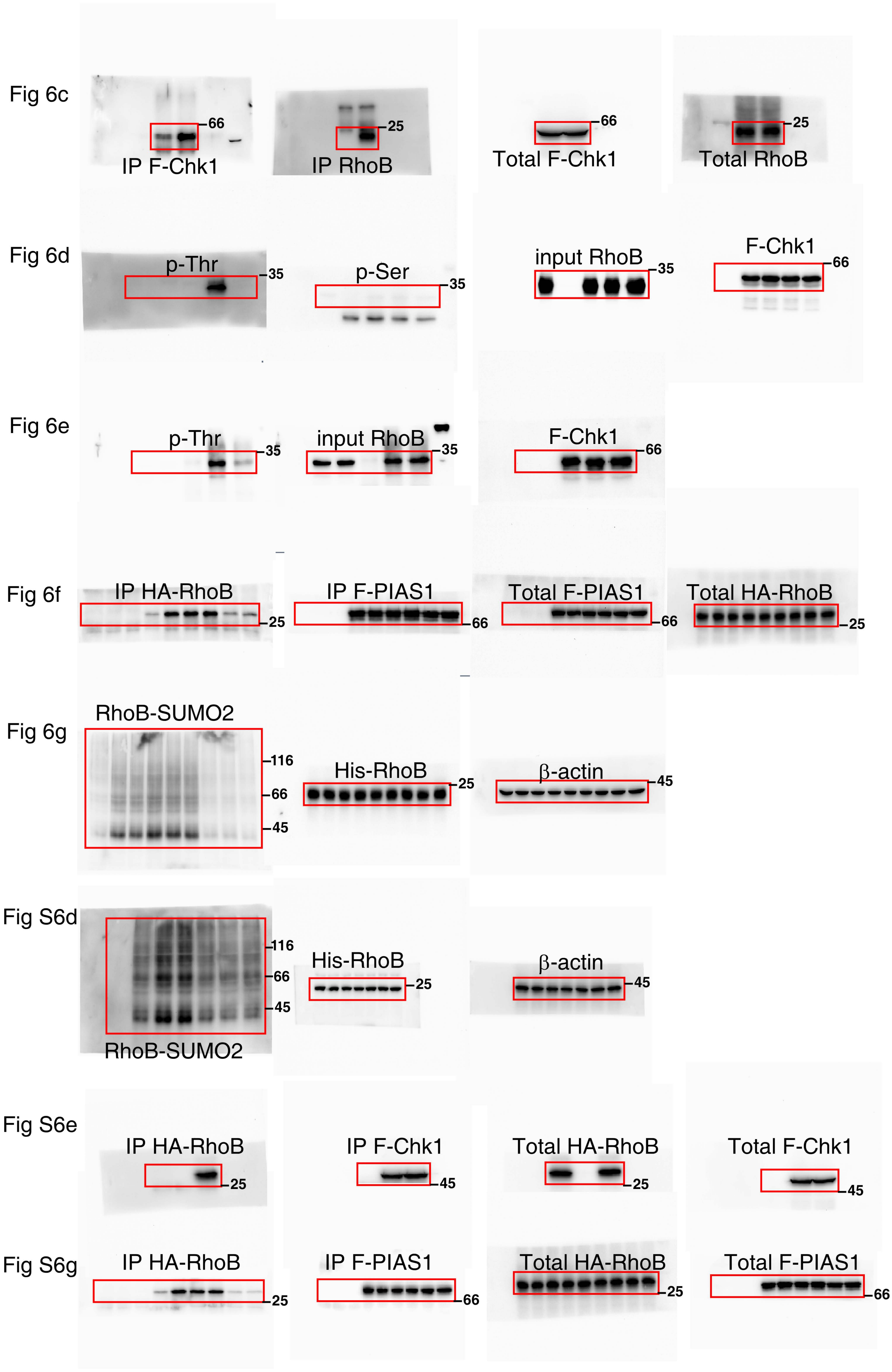

Fig 6c
IP F-Chk1
66
IP RhoB
25
Total F-Chk1
66
Total RhoB
25
Fig 6d
p-Thr
35
p-Ser
35
input RhoB
35
F-Chk1
66
Fig 6e
p-Thr
35
input RhoB
35
F-Chk1
66
Fig 6f
IP HA-RhoB
25
IP F-PIAS1
66
Total F-PIAS1
66
Total HA-RhoB
25
Fig 6g
RhoB-SUMO2
116
66
45
His-RhoB
25
β-actin
45
Fig S6d
RhoB-SUMO2
116
66
45
His-RhoB
25
β-actin
45
Fig S6e
IP HA-RhoB
25
IP F-Chk1
45
Total HA-RhoB
25
Total F-Chk1
45
Fig S6g
IP HA-RhoB
25
IP F-PIAS1
66
Total HA-RhoB
25
Total F-PIAS1
66

Fig 7a

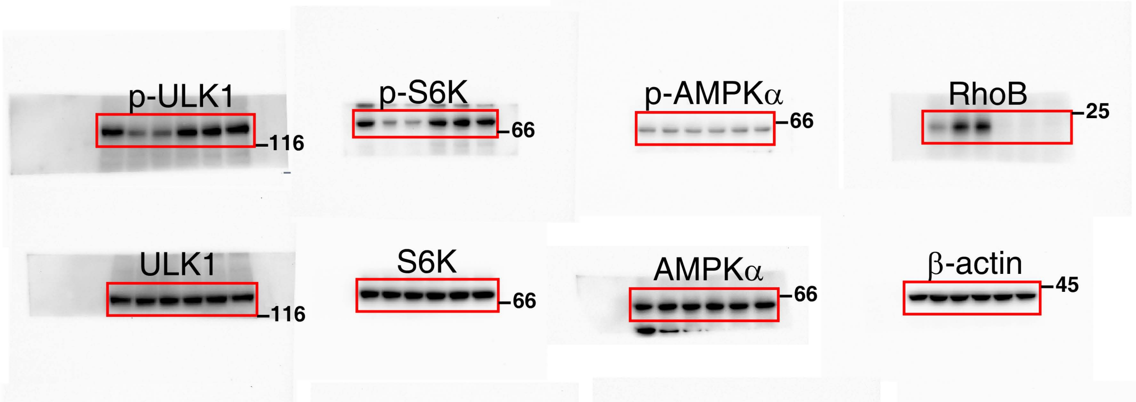

Fig 7b

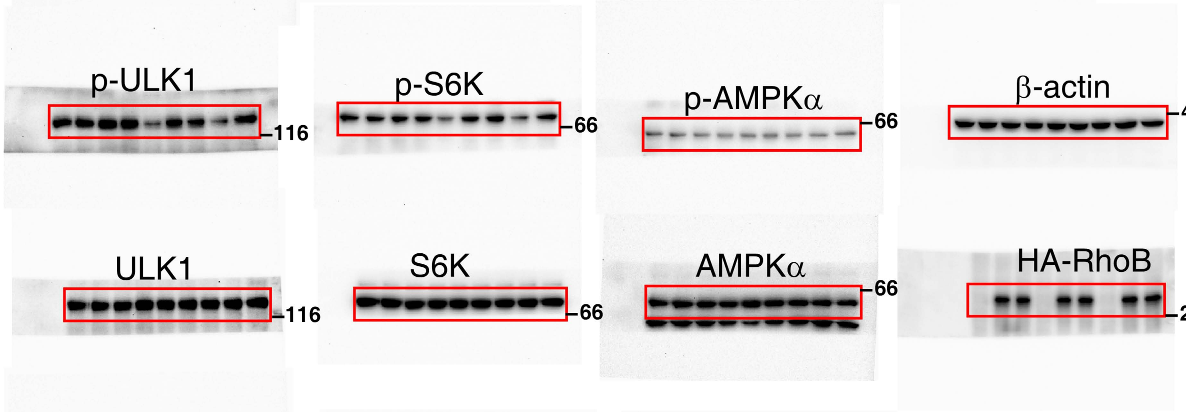

Fig 7f

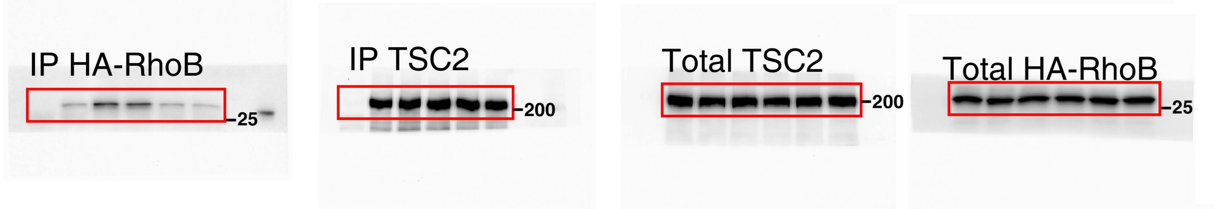

Fig 7g

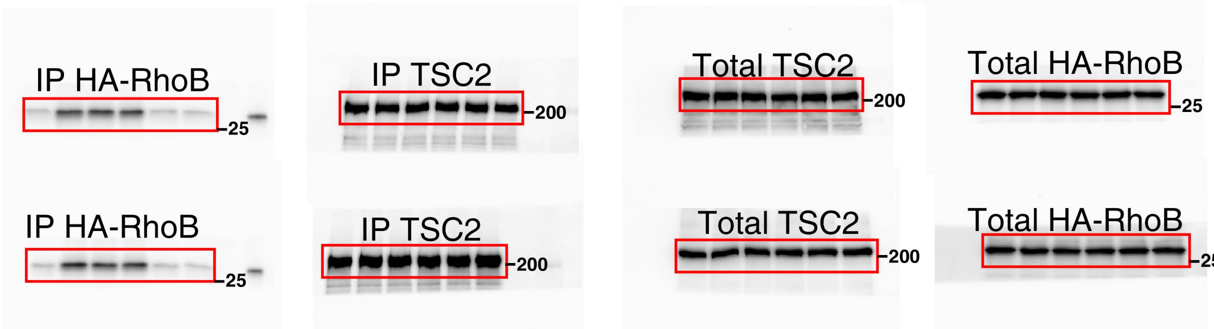

Fig 7h

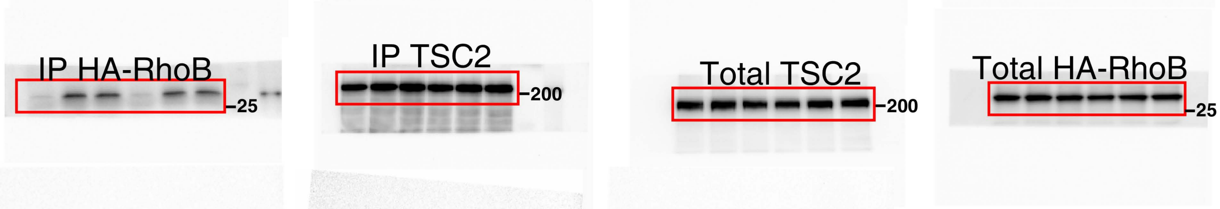

Fig S7a

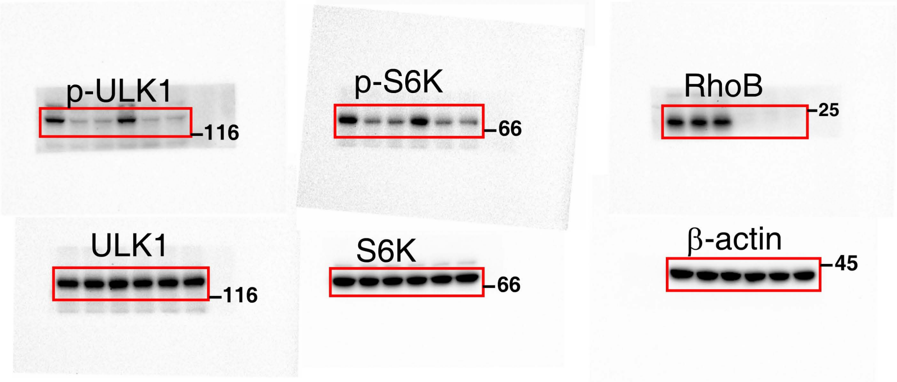

Fig S7b

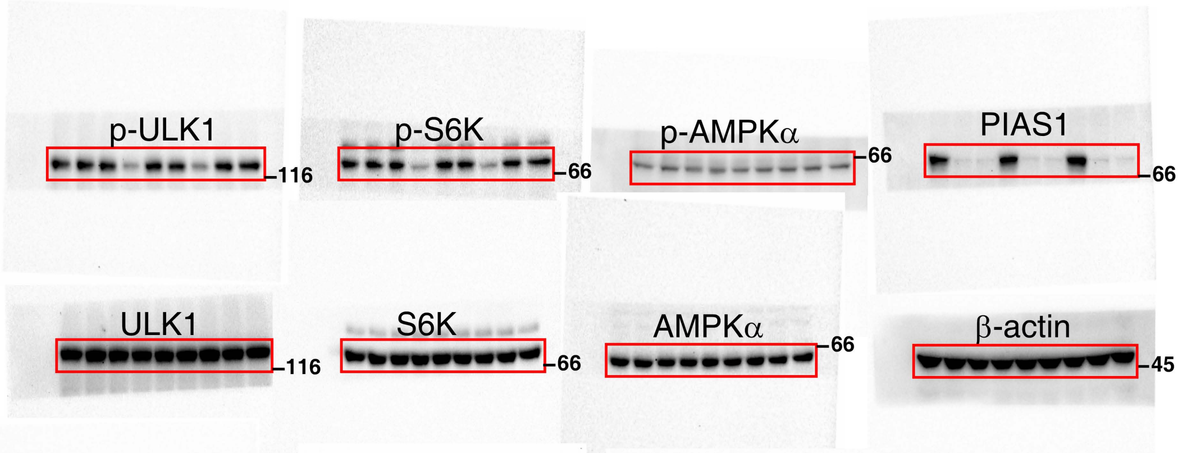

Fig S7e

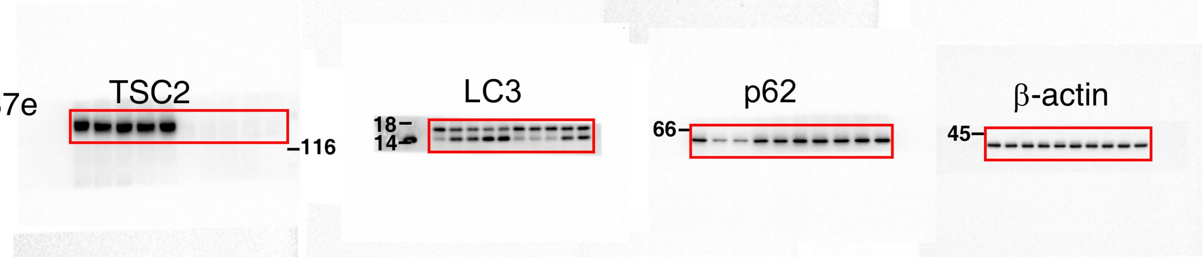

Fig S7i

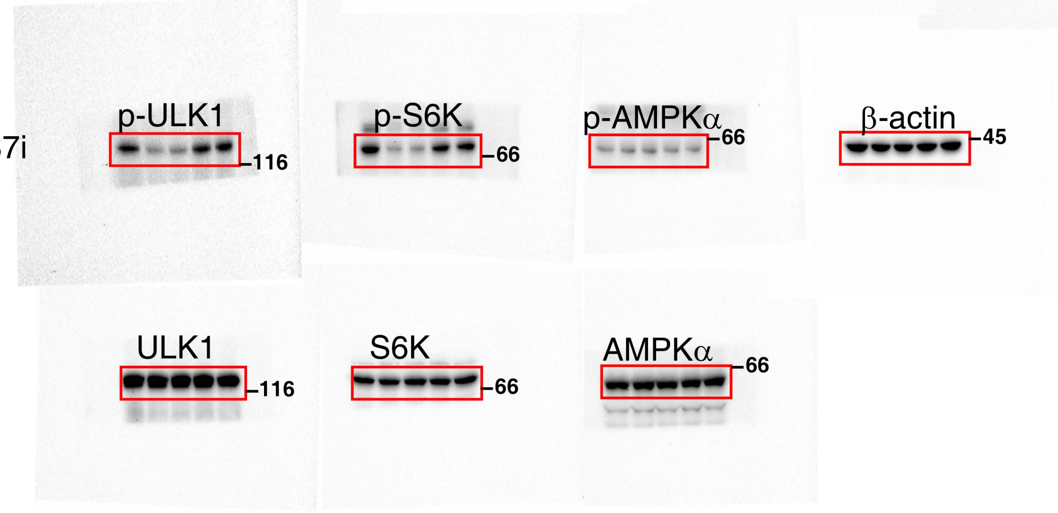

Supplement: Supplementary file 1 — Supplementary Information [file 41467_2018_6556_MOESM1_ESM.pdf]
